# Medical Ministry

**Published:** 1883-09-20

**Authors:** 


### *MEDICAL MINISTRY.*

[*Concluded from last month.*]

In the ministry of medicine the physician is the servant of the people. Yet, at the same time, he is master of the situation; for, under God, he holds the people's lives, as it were, in his hands, and to him they look for protection and defence against the dreaded and hydra-headed monster, Disease, and his all-conquering ally, Death.

But, to be vested with this power—with these sacred prerogatives, the minister of medicine must be a first-class man in every respect—first-class in his moralities, his character, acquirements and his skill. We should have Chevalier Bayards in medicine as well as in military and political circles, men without fear or reproach, and who do not consider any attainment too great to acquire in the grand and momentous work of adapting medical science and practice to the rescue of the world's sufferers from disease and the tortures of a lingering death.

It has been said that the commission of one unworthy man to the practice of medicine, was a direct means of demoralization. And why? Because the practice of medicine—and we say it with pride and gratification—the practice of medicine is a moral work. No science, especially one of such superior elements as that of medicine, can have a basis independent of an elevated, moral tone far superior to man—far above the finite conception of the human mind.

Not to recognize this unseen, but producing and propelling power of Omnipotence behind the laws that govern and interpret the science of medicine, is to ignore the presence of Nature and of Nature's God in that science, which is a most illogical and unscientific position to assume, and is also calculated to lower the tone of the whole medical profession and prostitute to base uses the noblest art of earth—that of healing the bodily ills of mankind.

As the character of no man is considered rounded and complete without the symmetrical finish of a moral development; so, the statue of the profession he represents will be moddeled in accordance with the exalted tone of his own ideas and desires. Then if the moral character of every physician should advance to completion side by side with the grand possibilities of progressive medicine, he would be truly fitted and equipped for the high position of his calling, for I can but believe that there is a time in the future when the minister and the interpreter of medicine will understand and master the laws that govern the human organization, he will be able to preserve these caskets of marvellous workmanship committed to his care by the great LAW-GIVER, and that contains the mystery of the still more marvellous soul of man. I can but believe the physician will yet be able to preserve these caskets in their natal strength and beauty, and resign them at the flat of death, only when man has lived out in both mental and physical vigor the full length of his allotted days

The Shekinah of medicine will have burst in all the refu'gence of its glory upon the medical world, and the physician will be known, loved and honored as the truest and best benefactor of the human race—without a fear, and as earth's best boon in the ranks of men. T. S P.

